# Is solute movement within the extracellular spaces of brain gray matter brought about primarily by diffusion or flow? A commentary on “Analysis of convective and diffusive transport in the brain interstitium” Fluids and Barriers of the CNS (2019) 16:6 by L. Ray, J.J. Iliff and J.J. Heys

**DOI:** 10.1186/s12987-019-0141-x

**Published:** 2019-07-12

**Authors:** Stephen B. Hladky, Margery A. Barrand

**Affiliations:** Department of Pharmacology, University of Cambridge, Cambridge, CB2 1PD UK

## Abstract

Solutes can enter and leave gray matter in the brain by perivascular routes. The glymphatic hypothesis supposes that these movements are a consequence of inward flow along periarterial spaces and an equal outward flow along perivenous spaces. The flow through the parenchyma between periarterial and perivenous spaces is the same as the inflow and the outflow. Ray et al. (Fluids Barriers CNS 16:6, 2019) have investigated how this flow could interact with diffusion using numerical simulations of real-time iontophoresis experiments that monitor the concentrations of tetramethylammonium ions (TMA^+^) injected into the parenchyma via iontophoresis. For this purpose they have devised a description of the parenchyma incorporating perivascular spaces. Their simulations show that superficial flow velocities of about 50 µm min^−1^ are needed to produce changes in TMA^+^ fluxes comparable to those accounted for by diffusion. In the glymphatic hypothesis the proposed flow through the parenchyma can be estimated from the clearance of solutes that are present in the perivenous outflow at the same concentration as in the interstitial fluid of the parenchyma. Reported clearances are approximately 1 µL min^−1^ g^−1^. This flow can be converted to a superficial flow velocity using the area available for the flow, which can be estimated using Ray et al.’s description of the tissue as 40 cm^2^ g^−1^. The best available estimate of the flow velocity is thus 0.25 µm min^−1^ which is 200 times smaller than the flow that produces effects comparable to diffusion for TMA^+^. Thus it follows in Ray et al.’s description of the parenchyma that diffusion rather than flow accounts for TMA^+^ movements. Because the diffusion constant depends only weakly on molecular weight the same is expected to apply even for solutes somewhat larger than serum albumin.

## Background

There is general agreement that solutes can enter and leave gray matter in the brain parenchyma via perivascular spaces, and that the rates of movement through these spaces are too fast to be mediated by diffusion alone (see [1, 2] for discussion and references). The glymphatic hypothesis explains these relatively rapid perivascular movements by proposing that solutes enter via fluid inflow along periarterial spaces and leave via a matching fluid outflow along perivenous spaces [3, 4]. The flow through the parenchyma is the same as the inflow and outflow. The question considered here is whether solutes move between the periarterial and perivascular spaces in the parenchyma primarily by diffusion or as a result of the flow.

## Main text

The proposed outflow and hence the flow through the parenchyma can be estimated from the clearance of a marker present at the same concentration in the outflow as in the extracellular fluid of the parenchyma (see section 3.2 in [2]). Clearances for a number of extracellular fluid markers like inulin have been reported to be about 1 µL min^−1^ g^−1^ (see Table 1 in [2]) which is currently the best available estimate of the flow required by the glymphatic hypothesis.

In a welcome and important advance Ray et al. [5] have investigated how the flow envisaged in the glymphatic hypothesis could interact with diffusion under conditions of real-time iontophoresis (RTI) experiments. Such experiments monitor the time-course of the concentration of an extracellular space marker, usually tetramethylammonium ions (TMA^+^), injected into the parenchyma via iontophoresis (see [6–8]). The TMA^+^ concentration is measured as a function of time using an ion selective electrode inserted a known distance, typically ~ 150 µm, from the site of injection. These time-courses are then fitted with the predictions of diffusion theory. The shape of the responses is as predicted by the theory. However, it is notable that (a) there is a substantial variation between the maximum concentrations that are measured in repeated experiments, and (b) it is difficult to assess the effects that flow might have using a theory that assumes there are none.

Ray et al. have devised a theoretical description of the gray matter in the brain capturing many of the essential features of the proposed flow between the perivascular spaces surrounding arterioles and venules but still simple enough to allow numerical simulations of RTI experiments. These simulations show that flow alters the concentrations that will be measured from one RTI experiment to the next. This occurs because the measuring electrode may be placed upstream of the iontophoresis electrode with the flow opposing the effects of diffusion or downstream with the flow augmenting the effects. The size of the changes depends on the orientation of the electrodes with respect to the direction of flow and on the superficial flow velocity, which is the flow across a cross-sectional area divided by the area. Ray et al. found that superficial flow velocities of about 50 µm min^−1^ altered the simulated concentrations by about ± 10% relative to the concentrations predicted with no flow (compare Figs. 5 and 6a in [5]). This estimate of the superficial velocity required to see an effect on the measured concentrations and hence on the fluxes of TMA^+^ is in agreement with the estimate, ~ 40 µm min^−1^, for a Peclet number of 1 calculated by Nicholson and Hrabetova [8]. Ray et al. note that flow with superficial velocity 50 µm min^−1^ combined with estimates of other sources of variation between experiments allows the data for repeated RTI experiments to be fitted.

An important question that arises is whether these superficial velocities inferred from the simulations are comparable to the superficial flow velocity in the parenchyma that can be calculated from the flow through the parenchyma and the area across which the flow occurs. The best available estimate for the flow, probably an upper limit for the actual flow, is that which accounts for the clearance of solutes like inulin in the glymphatic hypothesis. As indicated above, that estimate is ~ 1 µL min^−1^ g^−1^. The area can be estimated using Ray et al.’s model of the parenchyma and perivascular spaces. Ray et al. do not state this area, but they do say that 1 µL min^−1^ g^−1^ corresponds to 10 µm min^−1^ which implies that they have used 1 cm^2^ g^−1^ as the area. However, calculation of the area from the geometry of the perivascular spaces in their model leads to a different answer.

In Ray et al.’s description of the parenchyma the arterioles and venules are each assumed to be rods aligned in alternating sheets, i.e. on each side of a sheet or arterioles there is a sheet of venules and on each side of a sheet of venules there is a sheet of arterioles. The sheets are separated by 250 µm. The area available for flow between arterioles and venules in a gram of tissue is then the volume of tissue divided by the separation of the sheets, i.e. 1 cm^3^ g^−1^/250 µm = 40 cm^2^ g^−1^. Using these values the best available estimate of the typical superficial velocity in the parenchyma is flow/area = 1 µL min^−1^ g^−1^/40 cm^2^ g^−1^ = 0.25 µm min^−1^.

(Technical note: There is no direct way to calculate the flow from a volume average of the superficial flow velocity. Because the velocity is a vector quantity that can point in opposite directions at different locations within a region, the average of the velocity over that region can be zero even though the flow from sources to sinks within it is not. By contrast, the flow from the sources, i.e. the periarterial spaces, to the sinks, i.e. the perivascular spaces, can be calculated as a vector surface integral of the superficial flow velocity over surfaces that enclose each of the sources once while excluding the sinks. For the present model this calculation is particularly simple as the midplane between the arterioles and venules separates the sources from the sinks, and the superficial velocity at this surface is perpendicular to it and always pointing away from the layer of periarterial spaces. Thus for the conditions portrayed in Figures 6a and 4b of [5], the surface integral is equal to the product of the area of the midplane between the arterioles and venules, 40 cm^2^ g^−1^, and the average of the superficial velocity over that area which from Fig. 4b is close to 50 µm min^−1^).

## Conclusion

The average superficial velocity over the mid-plane between the arterioles and venules that was found in Ray et al.’s simulations to produce clear effects on the movement of TMA^+^ in the parenchyma is more than 200 times larger that the average velocity across the mid-plane that accounts for the clearance of solutes in the glymphatic hypothesis. To the extent that their model is an adequate description of the parenchyma there are two important consequences. Firstly it is unlikely that flow through the parenchyma as envisaged in the glymphatic hypothesis is an important part of the explanation of the variation in results between RTI experiments. Secondly, and much more importantly, because the simulations show that the flows which might exist in the parenchyma produce negligible changes in the concentrations and hence fluxes of tetramethylammonium ions, they provide further support for the commonly held view that extracellular solute movements in gray matter occur by diffusion. Because the diffusion constants for solutes vary only weakly with molecular weight (compare Tables 2 and 3 in [9] and discussion in section 4.1 and appendix C in [2]), this conclusion is likely to hold even for solutes as large as serum albumin.

It is important to note that the calculations in this commentary do not say anything useful about the processes moving solutes along perivascular routes.

## Data Availability

Not applicable.

## Abbreviations

RTI: real-time iontophoresis
TMA^+^: tetramethylammonium ions
